# Supplementary material for: Curated and harmonised transcriptomics datasets of interstitial lung diseases
Source: Data Brief. 2025 Oct 14;63:112139. doi: 10.1016/j.dib.2025.112139 (PMC12581653; doi:10.1016/j.dib.2025.112139)
Supplement: Supplementary file 1 [file mmc1.zip › Supplementary_material/RNA-seq/fastQC reports/GSE184316/SRR15926741_fastqc.html]

SRR15926741.fastq.gz FastQC Report 

FastQC Report

Mon 5 Feb 2024  
SRR15926741.fastq.gz

## Summary

- Basic Statistics
- Per base sequence quality
- Per sequence quality scores
- Per base sequence content
- Per sequence GC content
- Per base N content
- Sequence Length Distribution
- Sequence Duplication Levels
- Overrepresented sequences
- Adapter Content

## Basic Statistics

| Measure | Value |
| --- | --- |
| Filename | SRR15926741.fastq.gz |
| File type | Conventional base calls |
| Encoding | Sanger / Illumina 1.9 |
| Total Sequences | 9038428 |
| Sequences flagged as poor quality | 0 |
| Sequence length | 25-375 |
| %GC | 49 |

## Per base sequence quality

## Per sequence quality scores

## Per base sequence content

## Per sequence GC content

## Per base N content

## Sequence Length Distribution

## Sequence Duplication Levels

## Overrepresented sequences

| Sequence | Count | Percentage | Possible Source |
| --- | --- | --- | --- |
| GGAAGACAGTGTGGCGATTCCTCAGGGATCTAGAACTAGAAATACCATTG | 83251 | 0.9210783114054788 | No Hit |
| ATTGATTCTTCCTACCCATGAGCATGGAATGTTCTTCCATTTGTTTGTAT | 61939 | 0.6852850960366117 | No Hit |
| GAAGGACCTCTTCAAGGAGAACTACAAACCACTGCTCAAGGAAATAAAAG | 59416 | 0.6573709499041205 | No Hit |
| ATAGCAGCATGATTTATAGTCCTTTGGGTATATACCCAGTAATGGGATGG | 57436 | 0.6354644856384318 | No Hit |
| GAAGGACCTCTTCAAGGAGAACTACAAACCACTGCTCAATGAAATAAAAG | 39129 | 0.4329182021475416 | No Hit |
| GGCATCAAAAGTGATGGACTGCCCATTGCTGGAGAAGACCTTCTCTCCTA | 35571 | 0.3935529496943495 | No Hit |
| AGACATCAAATCCTGCAGACAAGGGGAGCCCTCAGTCTGCAGGGCTCCAT | 35388 | 0.39152826133039953 | No Hit |
| CAGTGGGGCCACGAGCTGAGTGCGTCCTGTCACTCCACTCCCATGTCCCT | 32632 | 0.36103623329189544 | No Hit |
| CAATCCACTTGATCCCAACTCATCTCTCATTTATTTCGGCTTCTTTTATT | 29983 | 0.3317280394334059 | No Hit |
| ATAGCAGCATGATTTATAATCCTTTGGGTATATACCCAGTAATGGGATGG | 29428 | 0.3255875911165083 | No Hit |
| CACCTCCCCGAAGCTCAGGGAGAGCCCTGTTAGGGCCGCCTCTGGCCCTA | 28280 | 0.3128862673907454 | No Hit |
| AGGCTACACACTGAGGACTCTGTTCCTCCCCTTTCCGCCTAGGGGAAAGT | 25230 | 0.2791414613249118 | No Hit |
| CTTCATGATGTAGCAGCAGGTGCCAGGGGCTGGCTTGTAGGCGATCAGCA | 23940 | 0.2648690679396904 | No Hit |
| TACCACTGCCACCTTCTCCATCGGCTCCACTGGCCTCGTGGTGTATGACT | 23871 | 0.26410566085164366 | No Hit |
| TGATATGGTTCCTGGCAAGCCCATGTGTGTTGAGAGCTTCTCAGACTATC | 23712 | 0.26234650538788384 | No Hit |
| TTACAAAAGCAGAATAAAAGCGAAAAGAAATGAAAATGTTACACTACATT | 23250 | 0.25723499705922315 | No Hit |
| TGATGACACCCACCGCAACTGTCTGTCTCATATCACGAACAGCAAAGCGA | 20349 | 0.22513870774873682 | No Hit |
| GGGAAGCGGGGAATGTCTACGTGCGTATGCACGTGGCACTCTCTGCCCGA | 18963 | 0.20980418276275478 | No Hit |
| GGCAATGAGCGGTTCCGCTGCCCTGAGGCACTCTTCCAGCCTTCCTTCCT | 16138 | 0.17854874763620399 | No Hit |
| ATTGATTCTTCCTACCCATGAGCATGGAATGTTCTTCCATTTGTTTGTGT | 15029 | 0.16627891487325008 | No Hit |
| GTCCACGTCACACTTCATGATGGAGTTGAAGGTAGTTTCGTGGATGCCAC | 14974 | 0.16567040197698094 | No Hit |
| GCATGTGTCTTTATAGCAGCATGATTTATAGTCCTTTGGGTATATACCCA | 14387 | 0.15917590979316315 | No Hit |
| GGAAGACAGTGTGGCGATTCCTCAAGGATCTAGAACTAGAAATACCATTG | 13715 | 0.15174098858783852 | No Hit |
| GGCCATTTTCATGATATTGATTCTTCCTACCCATGAGCATGGAATGTTCT | 13103 | 0.14496989963298929 | No Hit |
| ATAGCAGCATGATTTATAGTCATTTGGGTATATACCCAGTAATGGGATGG | 11308 | 0.12511025147293311 | No Hit |
| ATCAAAGAAGAACATGTGATCATCCAGGCCGAGTTCTATCTGAATCCTGA | 11282 | 0.12482259083106044 | No Hit |
| GGAAGACAGTGTGGCGATTCCTCAGGGATCTAGAACTAGAAATACCATTT | 11207 | 0.12399280051796618 | No Hit |
| GGAAGACAGTGTGGTGATTCCTCAGGGATCTAGAACTAGAAATACCATTG | 11047 | 0.12222258118336506 | No Hit |
| ACAAGAAGTACATCAAAGATTACATGAAATCAATCAAAGGGAAACTTGAA | 10783 | 0.11930171928127326 | No Hit |
| CAATGGGGTACTTCAGGGTCAGGATGCCACGCTTGCTCTGGGCCTCGTCG | 10651 | 0.11784128833022733 | No Hit |
| ACGTTTACCTGTCCATGTCTTACTACTTTGACCGCGATGATGTGGCTTTG | 10053 | 0.11122509356715571 | No Hit |
| GGAAGACAGTGTGGCGATTCCTCAGGGATCTAGAACTGGAAATACCATTG | 9761 | 0.1079944432815087 | No Hit |
| ACCTCTCTCTGGGCTTCTATTTCGACCGCGATGATGTGGCTCTGGAAGGC | 9706 | 0.10738593038523957 | No Hit |
| CAGCATGTTCCCTCTCCTCATGAGATTGGTGAAGAAAGTATTTGGCAAAG | 9523 | 0.10536124202128955 | No Hit |
| CTCTCTTTCTGGCCTGGAGGCTATCCAGCGTACTCCAAAGATTCAGGTTT | 9516 | 0.10528379492540074 | No Hit |

## Adapter Content

Produced by FastQC (version 0.11.7)
